# Four novel variants identified in primary hyperoxaluria and genotypic and phenotypic analysis in 21 Chinese patients

**DOI:** 10.3389/fgene.2023.1124745

**Published:** 2023-04-17

**Authors:** Qing Xin, Yameng Dong, Wencong Guo, Xiangzhong Zhao, Zhiying Liu, Xiaomeng Shi, Yanhua Lang, Leping Shao

**Affiliations:** ^1^ Department of Nephrology, Qingdao Municipal Hospital, Qingdao, China; ^2^ Medical Research Center, The Affiliated Hospital of Qingdao University, Qingdao, China; ^3^ Renal Division, Peking University First Hospital, Beijing, China; ^4^ Department of Nursing, Qingdao Municipal Hospital, Qingdao, China

**Keywords:** primary hyperoxaluria, *AGXT* gene, *GRHPR* gene, *HOGA1* gene, vitamin B6

## Abstract

**Background:** Primary hyperoxaluria (PH) is a rare genetic disorder characterized by excessive accumulation of oxalate in plasma and urine, resulting in various phenotypes due to allelic and clinical heterogeneity. This study aimed to analyze the genotype of 21 Chinese patients with primary hyperoxaluria (PH) and explore their correlations between genotype and phenotype.

**Methods:** Combined with clinical phenotypic and genetic analysis, we identified 21 PH patients from highly suspected Chinese patients. The clinical, biochemical, and genetic data of the 21 patients were subsequently reviewed.

**Results:** We reported 21 cases of PH in China, including 12 cases of PH1, 3 cases of PH2 and 6 cases of PH3, and identified 2 novel variants (c.632T > G and c.823_824del) in *AGXT* gene and 2 novel variants (c.258_272del and c.866-34_866-8del) in *GRHPR* gene, respectively. A possible PH3 hotspot variant c.769T > G was identified for the first time. In addition, patients with PH1 showed higher levels of creatinine and lower eGFR than those with PH2 and PH3. In PH1, patients with severe variants in both alleles had significantly higher creatinine and lower eGFR than other patients. Delayed diagnosis still existed in some late-onset patients. Of all cases, 6 had reached to end-stage kidney disease (ESKD) at diagnosis with systemic oxalosis. Five patients were on dialysis and three had undergone kidney or liver transplants. Notably, four patients showed a favorable therapeutic response to vitamin B6, and c.823_824dup and c.145A > C may be identified as potentially vitamin B6-sensitive genotypes.

**Conclusion:** In brief, our study identified 4 novel variants and extended the variant spectrum of PH in the Chinese population. The clinical phenotype was characterized by large heterogeneity, which may be determined by genotype and a variety of other factors. We first reported two variants that may be sensitive to vitamin B6 therapy in Chinese population, providing valuable references for clinical treatment. In addition, early screening and prognosis of PH should be given more attention. We propose to establish a large-scale registration system for rare genetic diseases in China and call for more attention on rare kidney genetic diseases.

## 1 Introduction

Primary hyperoxaluria (PH) is an autosomal recessive inherited disease resulting from abnormal hepatic glyoxylate metabolism, which is mainly characterized by excess oxalate production and excretion and progressive deposition of calcium oxalate in the kidney (Hoppe et al., 2009; Hoppe, 2012). The oversaturation of calcium oxalate can result in parenchymal inflammation, interstitial fibrosis, and urinary tract infection, and can, in some cases, lead to end-stage kidney disease (ESKD) and systemic oxalate deposition (systemic oxalosis) with time (Bobrowski and Langman, 2008; Shee and Stoller, 2022).

To date, three genetic forms of PH have been identified. PH1, the most prevalent and the most severe form, is caused by *AGXT* gene variants, which impair the function of hepatic alanine-glyoxylate aminotransferase (AGT). Consequently, excessive glyoxylate can’t be metabolized and was converted to oxalic acid, which was deposited in the kidney, resulting in recurrent urolithiasis and ESKD development during the early decades (Danpure and Jennings, 1986). Variants in the *GRHPR* gene inhibit the glyoxylate and hydroxypyruvate reductase (GR/HPR) activity and promote the occurrence of PH2. Compared with PH1, patients with PH2 generally have a less severe clinical picture, although recurrent urolithiasis may also lead to loss of renal function over time (Cramer et al., 1999; Cregeen et al., 2003). PH3 results from the deficiency of 4-hydroxy-2-oxoglutarate aldolase (HOGA), which is encoded by the *HOGA1* gene. It has been considered that PH3 was the least severe form with the maintenance of normal kidney function (Williams et al., 2012).

Until now, variants in the *AGXT*, *GRHPR* and *HOGA1* genes associated with PH have been described worldwide but rarely reported in China. The actual epidemiological and clinical characteristics of PH in Chinese populations remain unclear. Herein, this article described a series of 21 Chinese PH patients (12 of PH1, 3 of PH2, and 6 of PH3) with a wide clinical spectrum ranging from asymptomatic nephrolithiasis to ESKD and investigated their genotype-phenotype correlations.

## 2 Materials and methods

### 2.1 Patients

This study recruited 21 PH patients from 21 unrelated Chinese families who had been hospitalized in our nephrology department of Qingdao University Affiliated Qingdao Municipal Hospital from September 2017 to June 2022.

Among them, 10 were males and 11 were females. One patient (pt14) was born to first cousin parents. Necessary diagnostic criteria for PH included clinical findings (nephrocalcinosis, urolithiasis, end-stage renal failure, and other systemic complications), elevated levels of oxalate in urine (>0.5 mmol/1.73 m^2^/24 h), and confirmation of genetic testing. In addition, the elevated excretion of related biomarkers contributes to the type-specific diagnosis of PH: glycolate elevation (>0.418 mmol/mmol Cr) suggests PH1, elevated glycerate (>0.284 mmol/mmol Cr) suggests PH2, and excretion (>0.004 mmol/mmol Cr) of 4-hydroxy-glutamate (HOG) are pathognomonic of PH3. Hyperoxaluria caused by gastrointestinal disease or other secondary causes was excluded.

This study protocol was approved by the Ethics Committee of Qingdao University Affiliated Qingdao Municipal Hospital. Guardians or parents of the 21 PH patients have signed informed consent.

### 2.2 Variant analysis

#### 2.2.1 High-throughput sequencing and pathogenicity prediction of novel variants

Genomic DNA was extracted from the peripheral blood of these probands and their family members by GenElute blood genomic DNA kit (Sigma, NA 2010). High-throughput sequencing was used to analyze the exon regions and flanking intronic regions of three genes in each patient (*AGXT*, *GRHPR*, and *HOGA1*) associated with PH. Generated reads were then aligned to the human reference genome (UCSC hg19) using the Burrows-Wheeler Aligner (University of California, Santa Cruz, CA, United States). With Variant Effect Predictor v83 and the dbNSFP (Database for Non-synonymous SNPs’ Functional Predictions) v3.1, the variant call file (VCF) containing these variants was annotated. To predict the pathogenicity of the novel missense variants, web-based programs (SIFT, PolyPhen-2, and Mutation Taster) were used. Swiss-Pdb Viewer was used to elucidate the crystallographic structure of the protein, followed by energy minimization of wild-type and mutant protein structures using NOMADRef.

#### 2.2.2 Sanger sequencing verification

The potential candidate variants identified by NGS were validated by Sanger sequencing in patients and their family members. The suspected candidate variant sites and their flanking regions were amplified by PCR, and direct Sanger sequencing was performed using ABI prism 3700 DNA analyzer (Applied Biosystems, CA, United States). When heterozygous deletion or insertion was suspected, the PCR product was subcloned into the PGEM-T Easy vector (A1360; Promega), and sequenced using T7/SP6 primers.

### 2.3 Phenotype analysis

Collect information including age of onset, chief complaint for hospitalization, and main symptoms and signs, such as dysuria, renal colic, urgency and frequency of urination and hematuria in the form of questionnaire. Their laboratory results and images were reviewed, such as serum and urinary biochemical indexes, urinary electrolytes excretion fraction, stone risk factors (urine oxalic acid, urine glycolic acid, etc.), computed tomography as well as ultrasound examination of the urinary system.

### 2.4 Treatment and follow-up

Patients must avoid diets high in oxalic acid and vitamin C. Primary treatment includes supportive care, dialysis and surgery. Additionally, except those who were already on dialysis at diagnosis, all PH1 patients were treated with VB6. The initial oral dose of vitamin B6 was 5 mg/kg body weight/day, gained 5 mg/kg body weight every 6 weeks, and the final dose up to 20 mg/kg body weight/day in the 24th week. The threshold level for the efficacy of vitamin B6 treatment was defined as a relative 30% reduction in urinary oxalic acid (UOx) excretion. Serum and urine biochemical indicators, nephrocalcification/nephrolithiasis progression, and systemic complications were regularly followed up. Routine follow-up was performed in 21 cases, with a median follow-up of 2.1 years (0.3–4.5 years).

### 2.5 Genotype and phenotype association analysis

To analyze a possible genotype/phenotype correlation in PH1, we categorized patients into two groups (severe genotype group and mild genotype group) and performed a comparative study on the laboratory data at first admission between them.

## 3 Results

### 3.1 Analysis of variants

As shown in Table 1, there were 12 cases of type I, 3 cases of type II, and 6 cases of type III among all patients. Eight of them were homozygotes, the remaining thirteen were compound heterozygotes. Both alleles of the corresponding pathogenic gene were detected variants inherited from parents in each of the 21 probands. The results of high-throughput sequencing demonstrated that 23 variations (13 in type I, 4 in type II, and 6 in type III) were detected in our study. Variants with the highest frequency in PH1 were c.823_824dup (5/24) and c.33dup (4/24), while in PH3 were c.834_834 + 1GG>TT (5/12) and c.769T>G (3/12).

**TABLE 1:** Pathogenic variants of primary hyperoxaluria that were identified in this study.

Patient number	gene	Allele 1				Allele 2			
		Exon	Nucleotide change	Amino-acid change	References	Exon	Nucleotide change	Amino-acid change	References
1	*AGXT*	1	c.107G>A	p. Arg36His	Williams et al. (2009)	6	c.614C>T	p. Ser205Leu	Mandrile et al. (2014)
2	*AGXT*	6	c.679_680+2del	splicing donor	Coulter-Mackie et al. (2001)	1	c.33dup	p. Lys12Glnfs*156	Monico et al. (2007)
3	*AGXT*	6	**c.632T>G**	p. Leu211Arg	This study	6	**c.632T>G**	p. Leu211Arg	This study
4	*AGXT*	1	c.33dup	p. Lys12Glnfs*156	Monico et al. (2007)	5	c.577del	p. Leu193Phefs*19	He et al. (2019)
5	*AGXT*	8	c.823_824dup	p. Ser275Argfs*38	He et al. (2019)	4	c.466G>A	p. Gly156Arg	Mandrile et al. (2014)
6	*AGXT*	1	c.33dup	p. Lys12Glnfs*156	Monico et al. (2007)	11	c.1079G>A	p. Arg360Gln	Williams et al. (2009)
7	*AGXT*	8	c.823_824dup	p. Ser275Argfs*38	He et al. (2019)	8	c.823_824dup	p. Ser275Argfs*38	He et al. (2019)
8	*AGXT*	1	c.2T>C	p. Met1Thr	Coulter-Mackie and Rumsby (2004)	6	c.679_680+2del	splicing donor	Coulter-Mackie et al. (2001)
9	*AGXT*	1	c.145A>C	p.M49L	Wang et al. (2016)	1	c.145A>C	p. Met49Leu	Wang et al. (2016)
10	*AGXT*	8	c.823_824dup	p. Ser275Argfs*38	He et al. (2019)	11	c.1161C>A	p. Cys387*	He et al. (2019)
11	*AGXT*	8	**c.823_824del**	p. Ser275Profs*56	This study	8	**c.823_824del**	p. Ser275Profs*56	This study
12	*AGXT*	1	c.33dup	p. Lys12Glnfs*156	Zhao et al. (2021)	8	c.823_824dup	p. Ser275Argfs*38	He et al. (2019)
13	*GRHPR*	8	c.866_867del	p. Val289Aspfs*22	Cregeen et al. (2003)	8	c.866_867del	p. Val289Aspfs*22	This study
14	*GRHPR*	3	**c.258_272del**	p. His87_Asp91del	This study	3	**c.258_272del**	p. His87_Asp91del	This study
15	*GRHPR*	2	c.139C>T	p. Arg47*	Garrelfs et al. (2019)	8**	**c.866-34_866-8del**	splicing acceptor	This study
16	*HOGA1*	6	c.834_834+1GG>TT	p. Val279Cysfs*35	Fang et al. (2019)	6	c.834_834+1GG>TT	p. Val279Cysfs*35	Fang et al. (2019)
17	*HOGA1*	6	c.834_834+1GG>TT	p. Val279Cysfs*35	Fang et al. (2019)	6	c.834G>A	p. Ala278=	Wang et al. (2015)
18	*HOGA1*	7	c.845G>A	p. Arg282His	Huang et al. (2022)	6	c.812G>A	p. Arg271His	Fang et al. (2019)
19	*HOGA1*	6	c.769T>G	p. Cys257Gly	Belostotsky et al. (2010)	6	c.769T>G	p. Cys257Gly	Belostotsky et al. (2010)
20	*HOGA1*	6	c.834_834+1GG>TT	p. Val279Cysfs*35	Fang et al. (2019)	7	c.907C>T	p. Arg303Cys	Monico et al. (2011)
21	*HOGA1*	6	c.769T>G	p. Cys257Gly	Belostotsky et al. (2010)	6	c.834_834+1GG>TT	p. Val279Cysfs*35	Fang et al. (2019)

Novel variants are in bold type. Intron is marked with * *.

Notably, 2 variants (c.632T>G and c.823_824del) in *AGXT* and 2 variants (c.258_272del and c.866-34_866-8del) in *GRHPR* have not been reported previously in the literature. In addition, we also found a probably novel missense variant c.20G>C in *HOGA1* gene from a PH1 patient (pt11). The details of the predictive analysis on the pathogenicity of the novel missense variants were listed in Table 2.

**TABLE 2:** Prediction of the pathogenicity of the novel missense variants.

Gene	Variant	Mutation Taster	PolyPhen-2	SIFT	Conservative prediction	gnomAD MAF (%)	ExAC MAF (%)	ACMG classification
*AGXT*	c.632T>G	disease causing	probably damaging	damaging	Completely conservative	0.000	0.000	Likely pathogenic
*HOGA1*	c.20G>C	disease causing	benign	damaging	Completely conservative	0.002	0.002	Likely pathogenic

**FIGURE 1:**
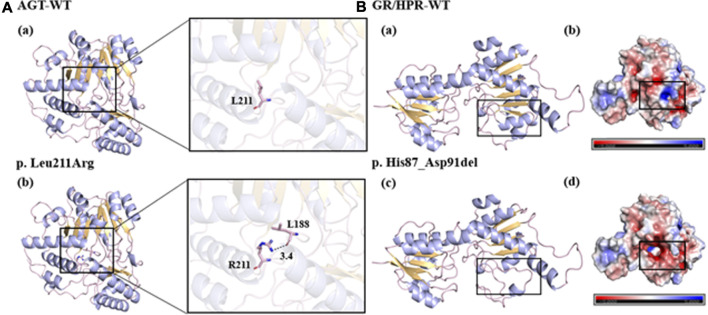
Predicted models of the novel variants. **(A-a, b, B-a, c)**: the bule line represented the target amino acid residue, the purple line represented the α helix, the yellow part represented the β layer, and the black dotted line represented the hydrogen bond. **(B-b, d)**: blue represented positive potential, white represented electrical neutrality, and red represented negative potential.

To investigate the potential impact of the novel mutants on protein folding and structure, three-dimensional (3D) structures of mutant protein were generated using Swiss-Pdb Viewer (Figure 1). With the change of amino acid sequence in AGT, the non-polar and hydrophobic leucine changed into alkaline arginine. In addition, the mutated R211 side chain formed a new hydrogen bond with the L188 main chain, which may have an adverse effect on the proper folding of the protein (Figure 1A). The deletion of amino acids from position 258 to 272 in GR/HPR destroyed the nearby α-helix and β-folding regions, and consequently, greatly changed the protein structure (Figure 1B-a, C). Meanwhile, the deletion of these amino acids produced new negatively charged cavities and replaced the original weakly negative and positively charged protein surfaces, which may affect protein folding or interaction with other molecules (Figure 1B-b, d).

### 3.2 Clinical manifestations and biochemical data

A total of 21 unrelated Chinese patients with PH were included in this study, and their basic information and biochemical results were summarized in Tables 3, 4. Median age at first clinical manifestation was 3 (interquartile range 1–10) years. Symptoms occurred before 1 year of age in 6 patients (30%) and after 18 years in 1 patient (5%). The median age at diagnosis was 4 (interquartile range 2–20) years. In the 15 patients who were symptomatic before 9 years of age, the mean diagnosis delay was 0.8 years (median 1.2 years). However, the remaining 6 patients (pt1, 6, 11, 12, 13, 14) who were symptomatic after 9 years had reached ESKD at diagnosis, and the mean diagnosis delay of them was 13 years.

**TABLE 3:** Clinical and biochemical characteristics of primary hyperoxaluria patients.

Patient number	Sex	Age at diagnosis (y)	Age at first onset (y)	Presenting symptoms	Urine calcium (mg/kg/24 h)	Plasma creatinine (μmol/l)		eGFR (ml/min/1.73 m2)	
1	female	35	22	Bilateral stones, UTI	5.07	88		123.6	
2	female	3.5	2	Bilateral stones, UTI, hematuria, hydronephrosis, dysuria	2.57	153		67.9	
3	male	4	2	Bilateral stones	1.99	85		112.3	
4	male	6.5	5	Bilateral stones, UTI, hematuria, dysuria	6.41	111		85.1	
5	female	3.2	3	Bilateral stones, UTI	1.77	95		94.1	
6	female	20	12	Bilateral stones, UTI	2.68	52		94.3	
7	male	4.0	1.0	Bilateral stones, UTI	2.31	45		96.8	
8	male	0.5	0.25	Bilateral stones, UTI	3.16	88		123.9	
9	female	0.3	0.25	Bilateral stones	2.97	92		101.5	
10	female	0.5	0.25	Bilateral stones, UTI, hematuria, hydronephrosis, dysuria	3.09	235		55.8	
11	male	25	18	Bilateral stones, dysuria	4.86	407		35.3	
12	male	18	10	Bilateral stones, UTI, hematuria	4.17	335		41.8	
13	male	32	11	Bilateral stones, hematuria	3.55	154		84.2	
14	male	30	9	Bilateral stones	2.79	76		92.5	
15	female	7.5	7	Bilateral stones, hematuria	3.36	103		79.4	
16	male	2.5	2.25	Bilateral stones, UTI	3.08	49		119.5	
17	female	5.5	5	Bilateral stones	4.51	53		120.7	
18	female	8	7	Unilateral stone	3.11	57		111.5	
19	male	0.5	0.3	Bilateral stones, UTI	7.55	70		121.3	
20	female	1	0.6	Bilateral stones, UTI	2.98	49		116.9	
21	female	2	1.5	Unilateral stone	4.14	82		95.2	

All data above were obtained at the first admission or before any treatment.

UTI, urinary tract infection; eGFR, estimated glomerular filtration rate.

**TABLE 4:** Analysis of urine organic acid components.

Patient number	Specific biomarkers (mmol/mmol Cr)			Urine oxalate (mmol/1.73 m2/24 h)	Urine oxalate after VB6 treatment (mmol/1.73 m2/24 h)*
	Urinary glycolate^a^	Urinary glycerate^b^	Urinary 4OHGlu^c^		
1	0.962	0.066	0.003	2.77	-
2	0.554	0.134	0.004	1.11	1.05 (5.4%)
3	0.497	0.214	0.002	0.86	0.76 (11.6%)
4	0.713	0.116	0.002	1.74	**1.16(33.3%)**
5	0.882	0.173	0.002	1.66	1.41 (15.1%0
6	-	-	-	3.12	-
7	0.534	0.238	0.003	0.96	**0.57(40.6%)**
8	0.627	0.098	0.004	1.08	**0.71(34.3%)**
9	0.725	0.187	0.002	1.41	**0.89(36.9%)**
10	0.768	0.183	0.003	1.55	1.46 (5.8%)
11	-	-	-	2.99	-
12	-	-	-	3.45	-
13	-	-	-	3.08	-
14	-	-	-	3.51	-
15	0.332	0.376	0.004	1.37	-
16	0.216	0.157	0.014	0.83	-
17	0.274	0.202	0.019	0.73	-
18	0.148	0.193	0.021	0.82	-
19	0.313	0.212	0.033	1.29	-
20	0.275	0.147	0.042	1.15	-
21	0.259	0.146	0.051	0.77	-

^a^ PH1 Specific biomarker.

^b^ PH2 Specific biomarker.

^c^ PH3 Specific biomarker.

*The data in brackets in this column indicates the percentage of urinary oxalate reduction after VB6 treatment.

Cr, creatinine; VB6, vitamin B6.

Common manifestations at initial visits in the 21 diagnosed PH patients were unilateral/bilateral stones (21/21), urinary tract infection (12/21), hematuria (6/21), dysuria (4/21) and hydronephrosis (2/21). None of the remaining family members presented any symptoms of hyperoxaluria or renal calculus. In addition, the mean levels of eGFR in the patients with PH1 (86 ml/min/1.73 m^2^) was lower than those in the patients with PH2 and PH3 (104.6 ml/min/1.73 m^2^), while the mean levels of creatine (148.8 umol/L) in PH1 was significantly higher than those in PH2 and PH3(77 umol/L).

Notably, patients who had reached ESKD presented various systemic complications, including difficult-to-correct hypoalbuminemia, severe anemia, hypercoagulation, optic atrophy, macular degeneration, decreased vision, glaucoma, hypothyroidism, and poor liver function with multiple abnormalities.

### 3.3 Treatment and follow-up

The treatment and outcomes of 21 patients are presented in Table 5. All patients received supportive therapy (high fluid intake, low-oxalate diet, etc.), and drug treatment including oral citrate and vitamin B6. Meanwhile, children (pt4, 17, 19) with hypercalciuria were also treated with oral hydrochlorothiazide. We observed the efficacy of VB6 in all PH1 patients and results showed that Vitamin B6 was effective in 4 patients (pt4, 7, 8, 9). The Uox levels of them decreased by an average of 36.3% after administration with VB6 (Table 4). In addition, most patients have undergone minimally invasive lithotripsy procedures, such as ureteroscopy, extracorporeal shock wave lithotripsy (ESWL), and retrograde intrarenal surgery (RIRS). Pt8 and pt9 were being maintained on citric acid and VB6 without any lithotripsy surgery, and have preserved intact renal function by now. Unfortunately, 5 patients (pt6, 11, 12, 13, 14) were on dialysis, which was not effective as expected.

**TABLE 5:** Management strategies and outcome of 21 patients.

Patient number	Procedures	Clinical outcome	Follow-up (years)	Urine oxalate (mmol/1.73 m^2^/24 h)	Plasma creatinine (μmol/l)	eGFR (ml/min/1.73 m^2^)
1	FURL, PCNL, KT	Stable	4.5	0.99	54	121.4
2	FURL, ESWL	Deteriorating	2.1	2.23	187	57.5
3	FURL	Deteriorating	2.8	1.01	89	78.2
4	FURL, RIRS	Stable	3.4	1.12	66	94.6
5	FURL, ESWL, LT	Stable	0.8	0.44	77	115.5
6	FURL, PCNL, HD	Deteriorating	1.8	-	257	-
7	FURL	Stable	2.5	0.61	45	124.9
8	-	Stable	0.5	0.72	64	111.3
9	-	Stable	1	0.85	68	97.4
10	FURL, LT	Stable	0.6	0.52	37	115.6
11	FURL, PCNL, HD	Deteriorating	2	-	312	-
12	FURL, PCNL, HD	Deteriorating	2.5	-	199	-
13	FURL, PCNL, HD	Deteriorating	4.2	-	267	-
14	FURL, PCNL, HD	Deteriorating	4.5	-	238	-
15	FURL, ESWL	Deteriorating	3.0	2.45	136	74.7
16	FURL, ESWL	Stable	1	0.77	52	119.3
17	FURL, RIRS	Stable	0.3	0.69	65	128.3
18	FURL, ESWL	Stable	1	0.86	75	116.3
19	FURL, ESWL	Stable	1	1.05	58	118.9
20	FURL, ESWL	Stable	0.5	1.11	60	123.7
21	FURL, RIRS	Stable	4	0.89	74	109.3

*FURL*, flexible ureteroscopy; *ESWL*, extra-corporeal shock wave lithotripsy; *RIRS*, retrograde intrarenal surgery, *PCNL*, percutaneous nephrolithotomy; *LT*, liver transplantation; *KT*, kidney transplantation; *HD*, hemodialysis.

What’s more, pt1 first presented with stones at the age of 22, and received a kidney transplant after diagnosis at the age of 35. Pt5 and pt10 have received liver transplantation, and both of them have acceptable renal function now. During follow-up, the urinary stones in 5 patients (pt1,4, 5, 9, 10) were completely removed without recurrence, and the remaining 16 patients showed recurrent urinary tract stones.

### 3.4 Genotype and phenotype association study

In general, the most severe phenotype was commonly observed in patients with non-sense, frameshift, canonical ±1 or 2 splice sites variants, single-exon or multiexon deletion, and loss-of-function missense mutants. Furthermore, since AGT functions as a homodimer, the residual activity of AGT may correspond to the residual activity resulting from a variant on one of the alleles. Therefore, we categorized patients into two groups: patients carrying 2 severe variants mentioned above on both alleles were classified as severe genotype group (pt2, 4, 6, 7, 10, 11, 12), while the others were assigned to mild genotype group (pt1, 3, 5, 8, 9). The results demonstrated that there was no significant difference in the age of onset (6.9 *versus* 5.5 years) between the two groups, however, significant higher creatinine (191.1 *versus* 89.6umol/L) and lower eGFR (68.1 *versus* 111.1 ml/min/1.73 m^2^) were detected in the severe genotype group than in the mild genotype group.

Notably, among the four patients (pt4, 7, 8, 9) who showed a favorable reactivity to vitamin B6 treatment, pt7 and pt9 were homozygous for c.823_824dup and c.145A>C respectively, while pt4 and pt8 were compound heterozygotes with different severe variants.

## 4 Discussion

### 4.1 Genotype and genotype-phenotype associations analysis of PH1

In the previous studies, the most frequent PH1 variants in the Chinese population were c.823_824dup, c.33dup and c.679-680 + 2del (Coulter-Mackie and Rumsby, 2004; Coulter-Mackie et al., 2008; Du et al., 2018; Li et al., 2018; He et al., 2019; Lin et al., 2021; Zhao et al., 2021). In our study, pt7 was homozygous for the variant c.823_824dup and lived with stable disease. This differed from previously reported conclusions, which suggested that this variant site was associated with a poorer prognosis (Zhao et al., 2021). So far, the variant c.33dup (p. K12Qfs) was documented as the second most common *AGXT* variant in the Chinese population (van Woerden et al., 2003; Monico et al., 2007; He et al., 2019; Zhao et al., 2021). Variant c.679-680 + 2del has been reported to have apparent ethnic associations with Chinese PH1 patients (Coulter-Mackie and Rumsby, 2004; Coulter-Mackie et al., 2008). It is located in the exon 6/intron 6 splice junction and is highly likely to interfere with the normal splicing process, as it is critical for initiating spliceosome assembly, precise selection of cleavage sites and interaction with U1snRNA and U6snRNA (Krawczak et al., 1992; Coulter-Mackie et al., 2001).

We identified a novel variant c.632T > G (p. Leu211Arg) in *AGXT* and pt3 was homozygous for it. The patient was diagnosed at the age of 4 and presented with classic recurrent urolithiasis with an increasing trend in creatinine in recent years. What’s more, this novel variant was located in the exon 6 of the *AGXT* gene, which spanned the common Pyridoxal 5′-phosphate (PLP) cofactor binding site consensus sequence (amino acids 201–221) of transaminases and was critical for the catalytic site (Ouzounis and Sander, 1993). Crystallization studies confirmed that the lysine at codon 209 was the actual site of the Schiff base and PLP (Zhang et al., 2003). Previous studies have identified more than a dozen variants on this motif (Nishiyama et al., 1991; Monico et al., 2005; Coulter-Mackie and Lian, 2006; Williams et al., 2009; Li et al., 2014). Therefore, variant c.632T > G may reduce or even abolish the catalytic activity of AGT by interfering with the binding of cofactors. The variant c.145A > C (p.M49L) was first reported in our previous study. Combined with software prediction results and clinical phenotype, it was considered to be “moderately pathogenic” at that time, and whether it had “true” pathogenicity remained to be confirmed (Wang et al., 2016). In this study, pt9, who was homozygous for this variant, showed typical symptoms at 3 months of age and was diagnosed as PH1 in infants. Therefore, the pathogenicity of this variant was further confirmed.

There was significant heterogeneity in clinical manifestations and laboratory examinations in PH1. Among the variants in this study, the variants p. Ser205Leu (<3% or<1%) and p. Arg360Gln (<1%) have been confirmed to cause almost loss-of-function of AGT through *in vitro* activity experiments, while the variants p. Arg36His (8.5%) and p. Gly156Arg (9%) have been confirmed to have some residual AGT activity (Coulter-Mackie and Lian, 2006; Williams et al., 2009). In addition, variant p. Met1Thr has been reported to be possibly associated with a milder clinical phenotype (Li et al., 2014; Zhao et al., 2021). Although it is located in the region of the start codon, it is possible that other downstream Mets act as alternative initial codons, resulting in the synthesis of AGTs with different N-terminus. Based on the work we performed, severe genotype group were found with higher creatine and lower eGFR, which indicated that genotype may influence the clinical presentation of PH1. In a sense, differences between the two groups suggested that there may be still some variations in the mild group that can confer AGT activity, which requires further functional expression research. Moreover, the ultimate phenotype and prognosis of patients may be affected by the age of diagnosis and clinical intervention, environmental factors and other variants from modifying genes, and just because of this, patients carrying the same variants may have great phenotypic heterogeneity (Mbarek et al., 2017).

In 1961, vitamin B6 was first described as a treatment choice for two patients with PH1(McLaurin et al., 1961). Pyridoxal 5′-phosphate (PLP) is a component of VB6 and a cofactor for AGT, which is defective in PH1. There are several different hypotheses about how PLP reduces endogenous oxalate production, such as increasing AGT enzymatic activity and stability, facilitating proper targeting of AGT to peroxisomes (Hopper et al., 2008; Fodor et al., 2012; Oppici et al., 2012; Oppici et al., 2015). A cohort study in Europe reported that response to VB6 treatment seemed to depend on potential variation (Mandrile et al., 2014). A prospective study reported that approximately 50% of patients showed a >30% reduction in Uox but did not reach complete normalization, even not in patients homozygous for the p. G170R variant (Hoyer-Kuhn et al., 2014). Notably, vitamin B6 treatment was also effective for some severe genotypes in our study, which may be associated with the residual AGT activity (Wanders et al., 1988). However, the efficacy of VB6 may not entirely depend on genotype, but also on differences in its absorption and metabolism among patients (Hoppe and Martin-Higueras, 2022). Furthermore, in addition to VB6, adjuvant therapy (such as citric acid supplementation), diet control compliance, and medicinal therapy can influence the outcome of treatment.

### 4.2 Genotype analysis in PH2 and PH3

Many findings have shown that the frequency of small deletions in PH2 was higher than expected (Cregeen et al., 2003). So far, two variants (c.139C>T and c.864_865del) in *GRHPR* gene from 2 patients have been reported in the Chinese population (Lam et al., 2001; He et al., 2019). In this study, we identified 4 variants from 4 patients, and 2 (c.258_272del and c.866-34_866-8del) of which were novel. The intronic variant c.866-34_866-8del is close to the 3′splice acceptor site of intron 8, and the pyrimidine content of the deleted sequence (CUU​AUC​UCC​CUC​UCU​CUC​UCU​CUC​UCC) is higher than 96%, which suggests that this sequence is likely to be located in the polypyrimidine tracts region in pre-mRNA. The absence of polypyrimidine tracts may interfere with the functioning of certain splicing factors and induce abnormal splicing (Auweter and Allain, 2008; Glasser et al., 2022).

It has been reported that the most common PH3 variant in the Chinese population was c.834_834 +1GG > TT, which accounts for approximately half of the PH3 variants identified in our study (Fang et al., 2019). In addition, the effect of consecutive substitution variant c.834_834+1GG > TT and synonymous variant c.834G>A on exon splicing has been verified in our previous mini-gene experiment (Wang et al., 2015). Interestingly, pt11 carried not only a homozygous *AGXT* gene variant c.823_824del, but also a heterozygous missense *HOGA1* variant c.20G > C. Regarding the pathogenicity of this variant c.20G > C, the results of different prediction software were not consistent. Therefore, further analysis and investigation are warranted to confirm whether c.20G>C serve as a modifying variant in the phenotype of the patient, or is a true PH3 pathogenic variant.

### 4.3 Underestimated prevalence of PH

Many studies have shown that the true proportion of PH in the renal stone and hyperoxaluria population is much higher than the observed prevalence (van der Hoeven et al., 2012; Garrelfs et al., 2019). In our study, 6 patients were severely delayed in diagnosis. Delayed diagnosis may impede early effective treatment and accelerate the occurrence of ESKD. The more serious possibility is that a large number of potential PH patients have not been diagnosed (Garrelfs et al., 2019). Therefore, it is necessary to reinforce screening for PH, and genetic testing is recommended for all patients with hyperoxaluria and recurrent stones. In addition, we suggest that a large registration system for rare diseases should be established in China to facilitate follow-up and instruct genetic counseling.

### 4.4 Treatment strategies for PH

So far, liver transplantation is the only established treatment for correcting metabolic defects that lead to the formation of excessive endogenous oxalates, but ideally before systemic complications occur (Devresse et al., 2020). For patients who have progressed to CKD IV (eGFR < 15–30 ml/min/1.73m2) and ineffective to vitamin B6 treatment without serious systemic oxalic acidosis, the first choice is combined liver and kidney transplantation (Murad and Eisenberg, 2017). However, transplantation is a morbid procedure associated with many potential complications, such as operative risks, graft rejection, post-transplant organ failure and the adverse effects of lifelong immunosuppressive medications (Asrani et al., 2018).

Hopefully, Lumasiran is a therapy based on RNA interference (RNAi), which can block the synthesis of oxalate glycolate oxidase and reduce the oxidation of glycolic acid to glyoxylic acid, thus avoiding further conversion of glyoxylic acid to oxalic acid (Hoppe and Martin-Higueras, 2022). Subcutaneous administration in animals shows 98% reduction in Uox (Liebow et al., 2017). Current experience with Lumasiran has been reported in both children and adults with PH1, with no specific side effects (Chiodini et al., 2021; Di Toro et al., 2021; Stone et al., 2021; Aldabek et al., 2022; Joher et al., 2022; Méaux et al., 2022). Good responsiveness has been shown in children. However, the effect is not significant in patients who already have systemic oxalic acidosis [37]. At present, clinical trials on the efficacy and safety of this therapy are under way, and it will be a promising treatment for PH in the future (Devresse et al., 2020; Michael et al., 2022).

### 4.5 Limitations of this study

There are certain limitations of this study. Firstly, the sample size of the study was insufficient, so the insights that can be provided were limited. In the future, it is necessary to carry further investigations with a larger sample size in the Chinese population, including polymorphisms and pathogenic variations. Besides, for some missense variations, more in-depth functional studies on activity are warranted. Furthermore, we suggest that studies should further confirm whether certain variations are really sensitive to VB6 treatment and how VB6 plays a therapeutic role.

To summarize, 4 novel variants in 21 Chinese PH patients were identified, including 2 in *AGXT* and 2 in *GRHPR* gene. A possible hotspot variant c.769T>G in PH3 was identified. Compared to PH1 patients, patients with PH2 and PH3 showed milder clinical phenotypes. Patients carrying severe variants in both alleles were vulnerable to more serious phenotypes. We found two potential VB6-sensitive genotypes, which would probably provide significant reference for clinical treatment. Delayed diagnosis still existed in some patients, and the actual prevalence of PH was likely to be much higher than what we have observed. Early screening of PH was very necessary, and intervention should be carried out as soon as possible to avoid the occurrence of ESKD.

## Data Availability

The original contributions presented in the study are included in the article/supplementary material, further inquiries can be directed to the corresponding authors.
